# Association between N-terminal Pro-Brain Natriuretic Peptide levels, glomerular filtration rate, and heart failure in the Multi-Ethnic Study of Atherosclerosis

**DOI:** 10.15761/jic.1000246

**Published:** 2018-05-22

**Authors:** Daneyal Syed, Stephanie Peshenko, Kiang Liu, Ramon Durazo-Arvizu, Sylvia E Rosas, Michael Shlipak, Mark Sarnak, David Jacobs, David Sickovick, João Lima, Richard Kronmal, Holly Kramer

**Affiliations:** 1 Department of Public Health Sciences, Loyola University Chicago, Maywood, IL, USA; 2 Medicine, Division of Nephrology and Hypertension, Loyola University Chicago, Maywood, IL, USA; 3 Department of Preventive Medicine, Feinberg School of Medicine, Northwestern University, Chicago, IL, USA; 4 Kidney and Hypertension Unit, Joslin Diabetes Center, Harvard Medical School, Boston, MA, USA; 5 General Internal Medicine Section, San Francisco Veterans Affairs Medical Center and University of California, San Francisco, USA; 6 Department of Medicine, Division of Nephrology, Tufts Medical Center, Boston, MA, USA; 7 Division of Epidemiology and Community Health, School of Public Health University of Minnesota, Minneapolis, USA and affiliated with the Department of Nutrition, University of Oslo, Oslo, Norway; 8 New York Academy of Medicine, New York, New York, USA; 9 Department of Medicine, Division of Cardiology, Johns Hopkins School of Medicine, Baltimore, MD, USA; 10 Collaborating health Studies Coordinating Center, University of Washington, Seattle, WA, USA

**Keywords:** chronic kidney disease, N-terminal pro-brain natriuretic peptide, heart failure, risk prediction, multi-ethnic study of atherosclerosis, estimated glomerular filtration rate

## Abstract

**Background:**

This study examined the complementary prognostic role of NT-proBNP and eGFR for predicting heart failure (HF) in adults with and without chronic kidney disease (CKD) defined as eGFR<60 ml/min/1.73m2.

**Methods:**

We used data from the Multi-Ethnic Study of Atherosclerosis, a cohort of 6814 adults without baseline clinical cardiovascular disease. Five-year risk prediction of HF based on clinical HF risk variables (HFRV) plus NT-proBNP, eGFR or both was assessed using the C-statistic and the net reclassification index (NRI) after stratifying by CKD status.

**Results:**

Mean age at baseline was 62.3±10.3 years and CKD were present in 5.9%. A total of 39 and 180 HF events occurred in participants with and without CKD, respectively. Among adults with CKD, the C-statistic for HF risk prediction increased significantly (P =0.04) from 0.71 (95% CI 0.64, 0.78) with HFRV alone to 0.78 (95% CI 0.71, 0.85) with addition of NT-proBNP. In the non-CKD group, the C-statistic increased from 0.77 (95% CI 0.74, 0.80) with HFRV alone to 0.83 (95% CI 0.80, 0.85) with addition of NT-proBNP. Further addition of eGFR to the model did not alter the C-statistic regardless of CKD status. NRI improved by 23.1% and 10.2% in CKD and non-CKD, respectively, with the addition of NT-proBNP alone and findings were similar when both eGFR and NT-proBNP were both added to model.

**Conclusions:**

In adults without clinical cardiovascular disease, the addition of NT-proBNP but not eGFR to established HFRV improves HF risk prediction in adults with and without CKD.

## Introduction

Presence of chronic kidney disease (CKD), defined as an estimated glomerular filtration rate (eGFR)<60mL/min/1.73m^2^, is associated with heightened risk of cardiovascular disease (CVD) including heart failure (HF) and this risk increases as eGFR declines [1–5]. Risk for incident HF is three-fold higher among adults with eGFR<60mL/min/1.73m^2^ *vs*. those with eGFR>90 [6]. Moreover, adults with eGFR<60mL/ min/1.73m^2^ who present with dyspnea are more likely to have HF compared to adults without CKD presenting with the same symptoms [7]. However, studies have not consistently demonstrated that eGFR improves HF risk prediction above and beyond established HF risk factors such as age, systolic blood pressure (SBP), body mass index (BMI), and heart rate (HR) [8–14].

N-terminal pro-brain natriuretic peptide (NT-proBNP) is a prohormone secreted by cardiac ventricles in response to excessive stretching of cardiomyoctes and has direct inhibitory effects on sodium transport in the inner medullary collecting duct [15] to help maintain volume homeostasis. Elevated levels of the biomarker NT-proBNP are strongly associated with HF [16–18] and are used clinically to diagnose HF in patients presenting with dyspnea [19–21]. However, higher NT-proBNP cut-points are required in settings of low eGFR [22] because NT-proBNP clearance is in part dependent on glomerular filtration rate [23–25].

Because both NT-proBNP and eGFR are associated with HF, the addition of NT-proBNP and eGFR with established HF risk factors may improve risk prediction for these outcomes, especially in adults with eGFR<60ml/min/1.73m^2^. A parsimonious HF risk score has been previously developed and validated and includes variables available to all clinicians: age, sex, smoking status, BMI, SBP, HR, and diabetes along with NT-proBNP [11]. This parsimonious HF risk score has a C-statistic of 0.87 for HF risk prediction in the Multi-Ethnic Study of Atherosclerosis (MESA) cohort, a group without baseline CVD, but eGFR was not examined as a potential variable. This study examines the complementary prognostic role of NT-proBNP and eGFR with established clinical HF risk factors for prediction of HF in adults. Analyses were stratified by presence of CKD defined as eGFR<60 ml/ min/1.73m^2^ due to the significant statistical interaction between CKD status and NT-proBNP levels on HF risk. We hypothesized that both eGFR and NT-proBNP will improve HF risk prediction in adults with CKD.

## Methods

### Study population

The Multi-Ethnic Study of Atherosclerosis (MESA) is a population-based study of 6,814 men and women aged 44–84 years, without clinical CVD, recruited from six U.S. communities between 12/1/2000 and 7/30/2002 (Baltimore, MD; Chicago, IL; Forsyth County, NC; Los Angeles County, CA; northern Manhattan, NY; and St. Paul, MN). Sampling and recruitment procedures have been described in detail elsewhere [26]. Subjects with symptoms or history of medical or surgical treatment for CVD were excluded at study entry. Adults who self-reported as non-Hispanic White, African-American, Hispanic, or Chinese were invited to participate. Institutional Review Board approval was obtained at all MESA sites. Of the 6,814 subjects, 5,597 had plasma NT-proBNP measurement and 88 participants were excluded due to missing data on at least 1 other MESA 5-year HF risk variables (age, sex, body mass index, smoking status, heart rate, systolic blood pressure and diabetes), cystatin C levels, or serum creatinine levels at the baseline examination. Five individuals (3 with CKD and 2 without CKD) were not followed for events, leaving a total of 5,504 participants included in the present analyses.

### Brain natriuretic peptide (NT-proBNP) measurements

NT-proBNP levels were measured using the Elecys-electrochemiluminescence immunoassay (Roche Diagnostics Corporation, Indianapolis, IN, USA). Analyses were performed at the University of California, San Diego using a 250μl serum sample drawn at the baseline examination and were previously unthawed or only thawed once. Intra- and inter-assay coefficients of variation (CV) were 2.7% and 3.2%, respectively, at 175pg/mL, 2.4% and 2.9%, respectively, at 355pg/mL, 1.9% and 2.6%, respectively, at 1068pg/mL, and 1.8% and 2.3%, respectively, at 4962 pg/Ml [27].

### Estimated glomerular filtration rate (eGFR)

Cystatin C measurements were made using a BNII nephelometer on plasma specimens (N Latex Cystatin C; Dade Behring Inc., Deerfield, IL). The assay range was 0.195 to 7.33 mg/L. The reported reference range for cystatin C among young, healthy individuals is 0.53 to 0.96 mg/L. Intra- and inter-assay CV ranges were 2.0 to 2.8%, and 2.3 to 3.1%, respectively. Serum creatinine was measured at exam 1 using colorimetry with a Johnson & Johnson Vitros 950 analyzer (Johnson & Johnson Clinical Diagnostics Inc. Rochester, NY). Creatinine values measured at exam 1 were calibrated with creatinine values measured at exam 5 using an isotope dilution mass spectrometry traceable method. The intra- and inter-assay CVs were <2%. Presence of CKD was defined as an eGFR<60mL/min/1.73m2 by the creatinine-cystatin C CKD-EPI equation [28].

### Heart failure risk variables (HFRV)

For the purpose of this analysis, we define HFRV as the variables identified within the MESA 5-year HF risk score (age, sex, smoking status, BMI, SBP, HR, and diabetes) with the exception of NT-proBNP. All MESA participants completed self-administered questionnaires in English, Spanish, or Chinese and were interviewed by trained research staff in order to collect information pertaining to demographic characteristics, medical history, medication, alcohol and tobacco use. Trained and certified clinic staff obtained blood pressure and anthropometric measurements on all MESA participants during the baseline visit. After a five-minute rest, blood pressure was measured three times at one-minute intervals using a Dinamap PRO 100 automated oscillometric device (Critikon; Tampa, FL) with the subject in a seated position with the back and arm supported. The average of the second and third SBP measurements was used for this analysis. Diabetes mellitus was defined as a self-reported diagnosis, use of insulin or oral hypoglycemic agents, or fasting glucose≥126mg/dL. Smoking status was dichotomized as current smoking versus former or never smoker.

### Heart Failure (HF)

Information on new cardiovascular events was obtained by a telephone interviewer who contacted each participant or family members every 9–12 months to inquire about interim hospital admissions, cardiovascular outpatient diagnoses, and deaths. Self-reported diagnoses were confirmed via death certificates and medical records for all hospitalizations and outpatient cardiovascular diagnoses. All cardiovascular events were adjudicated and classified by two members of the mortality and morbidity review committee (cardiologists or cardiovascular physician epidemiologists). Persisting disagreements were classified by the full review committee. Reviewers classified HF as definite, probable, or absent. A designation of definite or probable HF required HF symptoms, such as shortness of breath or edema. Additionally, classification of probable HF required a physician diagnosis of HF and documentation of medical treatment. Definite HF also required one or more additional criteria, such as pulmonary edema/congestion by chest X-ray, decreased left ventricular systolic function by echocardiography or ventriculography, or evidence of left ventricular diastolic dysfunction by echocardiography [29]. Definite or probable HF was used to define HF for the present analysis.

### Statistical analysis

Descriptive baseline characteristics of MESA participants were examined after stratifying by CKD status. Continuous variables were compared by CKD status using the unpaired t-test. Categorical variables were compared using the χ^2^ statistic. Spearman correlation coefficients were calculated to determine the linear association between eGFR and log transformed NT-proBNP in the MESA participants stratified by CKD status.

Cox proportional hazard models were used to calculate the hazard ratios for HF by presence of clinical HF risk variables, log transformed NT-proBNP and eGFR. The assumptions of the Cox proportional hazards models were examined by plotting the natural log of the cumulative hazard of HF by the natural log of time. The natural log transformation was applied to NT-proBNP to address the skewed distribution. A multi-model approach was used to examine the association between eGFR and NT-proBNP and hazard of incident HF while simultaneously adjusting for HFRV. Model 1 included HFRV: age, sex, BMI, smoking status, SBP, HR, and diabetes. Model 2 included the HFRV plus eGFR; Model 3 included the HFRV plus log transformed NT-proBNP, and Model 4 included HFRV, log transformed NT-proBNP and eGFR. A statistically significant interaction was noted between CKD status and log transformed NT-proBNP (P = 0.007) on the hazard rate of HF with adjustment for all covariates. Therefore, separate Cox proportional hazards models were constructed for those with and without baseline CKD. To compare risk discrimination, we calculated the area under the receiver operating characteristic (ROC) curve (C statistic) and likelihood-based measures (-2 Log Likelihood, the Akaike information criterion [AIC] and the Bayes information criterion [BIC]) for each model [30,31].

MESA participants were classified according to their 5-year HF risk: low risk (<5%), average risk (5–10%), and high risk (>10%) based on each model. Despite 12-year follow-up data, 5-year HF risk was calculated in accordance with the 5-year MESA HF risk score [11]. Risk stratification capacity was defined as the proportion of participants classified as high or low risk and who did or did not have an event, respectively, and net reclassification improvement (NRI) was calculated [32–34]. Individual components of the NRI, assessing improvement in event and non-event classifications, were calculated [33]. Z scores were calculated for the NRI and its individual components and hypothesis testing was conducted using an asymptotic test for the null hypothesis of NRI=0 [33]. Statistical analyses were performed with the use of STATA software version 14.2 (STATA Corp., College Station, Texas).

## Results

Overall, a total of 325 participants with CKD and 5,179 without CKD were included in the analyses. In the CKD group, 8.3% (n=27) had an eGFR<30 mL/min/1.73m^2^. The median duration of follow-up was 12.1 years (IQR: 11.6–12.7), and incidence rate of HF was higher among participants with CKD (13.1 [95% CI 9.6, 18.0] per 1000 person-years) than those without CKD (3.1 [95% CI 2.7, 3.6] per 1000 person-years, p<0.001). The characteristics of the MESA participants by CKD status are shown in Table 1. Participants with CKD were significantly older (73.0±8.0 *vs*. 61.6±10.1 years; p<0.001) and more likely to have hypertension (78.2% *vs*. 42.1%; p < 0.001) and diabetes (19.9% *vs*. 12.6%; p<0.001). Geometric mean values of NT-proBNP values were 158.0 pg/mL (95% CI 140, 178) in the CKD group and 47.2 pg/mL (95% CI 45.8, 48.7) in the non-CKD group.

Table 2 shows the characteristics of MESA participants with and without incident HF after stratifying by CKD status. A total of 39 and 180 HF events occurred in the CKD and non-CKD groups, respectively. Among those with CKD, participants with HF were more likely to be male (64.1% *vs*. 40.2%; p=0.005), have higher SBP (144±26.8 *vs*. 139±24.7 mmHg; p=0.16) and have diabetes (35.9% *vs*. 19.6%; p=0.02). Geometric mean values of log transformed NT pro-BNP levels were significantly higher among those with HF than those without HF in the CKD group (331 [95% CI: 215, 510] *vs*. 143 [95% CI: 127, 161] pg/mL; p<0.001). In the non-CKD group, participants experiencing events were significantly older (67.9±8.7 *vs*. 61.4±10.0 years; p<0.001), had greater BMI (29.5±5.6 vs 28.2±5.4 kg/m^2^; p=0.002), SBP (137±21.9 *vs*. 125±20.8 mmHg; p<0.001), heart rate (65.9±10.5 *vs*. 63.0±9.5 bpm; p<0.001), and geometric mean values of NT pro-BNP levels (126 [95% CI: 106, 149] *vs*. 45.6 [95% CI: 44.2, 47.0] pg/mL; p<0.001). Significant correlations were noted between log transformed NT-proBNP levels and eGFR among participants with CKD (r=−0.19; p<0.001) and without CKD (r=−0.33; p<0.001).

Results from the Cox proportional hazard models with HFRV, eGFR, and NT-proBNP for hazard of incident HF in MESA participants stratified by baseline CKD status are presented in Table 3. The continuous variable eGFR was not significantly associated with HF after adjustment for HFRV in the CKD or non-CKD groups. LogNTproBNP remained significantly associated with HF after adjustment for HFRV in both the CKD and non-CKD groups. Table 4 shows the measures of model fit for HF risk prediction with HFRV alone or when combined with eGFR, NT-proBNP or both. Regardless of the model, the C statistic was consistently lower in the group with CKD *vs*. without CKD (P < 0.01 for all models). Overall, the highest C-statistic and lowest AIC and BIC were noted for models that included NT-proBNP in the CKD and non-CKD groups. Further addition of eGFR to the model that included HFRV and NT-proBNP did not appreciably increase the C-statistic regardless of CKD status.

Reclassification analyses demonstrated that addition of NT-proBNP to HFRV improved NRI by 23.1% (p=0.07; pevents=0.53; pnon-events<0.001) in those with CKD and by 10.2% (p=0.003; pevents=0.01; pnon-events<0.001) in those without CKD while the addition of eGFR to HFRV changed the NRI by 6.53% (p=0.09; pevents=0.16; pnon-events=0.25) and −0.46% (p=0.72; pevents=0.65; pnon-events=0.45) in the CKD and non-CKD groups, respectively. The addition of both NT-proBNP and eGFR to HFRV improved net reclassification by 28.8% (p=0.02; pevents=0.40; pnon-events<0.001) in those with CKD and by 15.9% (p<0.001; pevents<0.001; pnon-events=0.54) in those without CKD (Table 5–6).

## Discussion

This study shows that the addition of NT-proBNP with HFRV improves HF risk prediction in adults with and without CKD compared to a HFRV model without NT-proBNP. Adding eGFR to the model with HFRV+NT-proBNP model slightly improves NRI in adults with or without CKD. Based on the Z scores for net reclassification, adding eGFR to a model which includes NT-proBNP and HFRV improves the risk classification of non-HF events in those with CKD and risk classification of HF events in those without CKD. However, the addition of eGFR with NT-proBNP and HFRV does not change the C-statistic or likelihood-based measures indicating eGFR does not increase overall risk prediction of HF.

To date, four major cohort studies have published HF risk scores [9,11,12,35]. The most recent risk score was developed in the MESA cohort. Creatinine itself was examined as a potential variable in the MESA HF risk score but was not included in the final proposed risk score; cystatin C and eGFR were not studied. We applied this MESA HF risk score to the MESA cohort stratified by CKD status and studied the complementary prognostic role of NT-proBNP and eGFR. Incidence of HF was higher among participants with CKD, and a significant and inverse correlation existed between eGFR and NT-proBNP levels among both CKD and non-CKD groups. We found that overall model fit based on the C-statistic was lower in MESA participants with CKD *vs*. those without CKD. This is consistent with other previous studies showing that cardiovascular risk prediction models do not perform as well in populations with CKD compared to populations without CKD [34,36].

The MESA study did not include individuals with baseline clinical cardiovascular disease. Flores and colleagues studied the prognostic value of cystatin C-based eGFR in patients with acute decompensated HF and found that the combination of NT-proBNP levels with cystatin C based eGFR predicted all cause death and HF readmissions better than either parameter alone [8]. Similarly, Van Kimmenade and colleagues observed that NT-proBNP combined with eGFR based on serum creatinine predicted 60-day mortality among patients with HF better than either parameter alone [10]. In both analyses, eGFR was dichotomized as <60 mL/min/1.73m2 and NT-proBNP was dichotomized along its median value.

In the acute setting, McCullough and colleagues observed a weak correlation between eGFR based on serum creatinine and B-type natriuretic peptide (BNP) [22]. In their study, eGFR was a significant and independent predictor of HF along with BNP, presence of a S3 gallop, and diabetes in a cohort of participants presenting to the emergency department with acute dyspnea. However, approximately one-third of the participants had a history of HF and BNP was much more influential in the risk prediction model than eGFR. Similarly, investigators examining the interaction between eGFR and NT-proBNP levels in the ProBNP Investigation of Dyspnea in the Emergency Department (PRIDE) Study found log-transformed NT-proBNP concentrations to be the strongest predictor of 60-day death even with the inclusion of eGFR in the model [37].

The strengths of this study include the adjudication of all outcomes and the standardized measures of HFRV. MESA also includes four racial/ethnic groups from six different sites across the United States. This study has several limitations. The observed effect of eGFR on risk prediction of incident HF may have been limited by the low number of events in the CKD group (n=39) and a narrow eGFR range with less than 10% of the adults with CKD having an eGFR<30ml/min/1.73 m^2^. It’s possible that eGFR may be more informative for predicting HF risk among adults with more advanced CKD. MESA also excluded individuals with morbid obesity limiting generalizability to many adults at high risk for HF. Our time period was limited to 5 years of follow-up because the MESA HF risk score was based on events over this time period.

## Conclusion

In conclusion, we found that NT-proBNP was a significant predictor of HF regardless of CKD status and the inclusion of eGFR alone did not substantially improve HF risk stratification compared to HFRV plus NT-proBNP. However, the addition of eGFR along with NT-proBNP to a model with traditional HFRV slightly improved HF risk reclassification compared to a model with HFRV plus NT-proBNP alone. Future studies should examine other biomarkers to improve risk prediction of HF in adults with and without CKD.

## Figures and Tables

**Table 1.** Baseline characteristics of the Multi-Ethnic Study of Atherosclerosis (MESA) participants by baseline Chronic Kidney Disease (CKD) Status

	Total (n=5,504)	CKD (n=325)	Non-CKD (n=5,179)	*P-value
Age (years)	62.3±10.3	73.0±8.0	61.6±10.1	<0.001
Sex (Male)	48.6%	43.1%	48.9%	0.04
Black	24.1%	22.8%	24.2%	0.6
White	39.4%	45.2%	39.0%	<0.001
Hispanic	23.1%	19.4%	23.4%	0.1
Chinese	13.4%	12.6%	13.5%	0.7
Smoking	12.6%	8.3%	12.9%	0.02
BMI (kg/m^2^)	28.2±5.4	28.5±5.4	28.2±5.4	0.3
Diabetes Mellitus	13.2%	19.9%	12.6%	<0.001
Heart rate (beats per minute)	63.1±9.7	63.6±11.2	63.1±9.6	0.3
SBP (mmHg)	126±21.4	139±25.0	126±20.9	<0.001
Hypertension	44.2%	78.2%	42.1%	<0.001
Blood pressure medication use	32.6%	64.6%	30.6%	<0.001
LDL (mg/dL)	117±31.4	116±33.5	117±31.2	0.74
HDL (mg/dL)	50.8±14.8	49.6±15.0	50.9±14.8	0.1
Cholesterol (mg/dL)	194±35.9	196±41.4	194±35.5	0.3
Lipid lowering medication use	16.1%	27.4%	15.4%	<0.001
†NT-proBNP (pg/mL)	50.7 (49.1, 52.3)	158.0 (140, 178)	47.2 (45.8, 48.7)	<0.001
Cystatin C (mg/L)	0.90±0.25	1.49±0.60	0.86±0.15	<0.001
Creatinine (mg/dL)	0.84±0.29	1.33±0.80	0.81±0.17	<0.001
Creatinine-cystatin C eGFR (mL/min/1.73m^2^)	89.8±18.4	48.8±11.0	92.4±15.5	<0.001
Heart failure	219 (3.98%)	39 (12.0%)	180 (3.48%)	<0.001

Data presented as mean ± standard deviation or frequency (%) unless otherwise noted.

* P-values compare CKD with Non-CKD groups. Abbreviations: CKD = chronic kidney disease; BMI = body mass index; SBP = systolic blood pressure; LDL = low-density lipoprotein; HDL = high-density lipoprotein;

† NT-proBNP = N-terminal pro-brain natriuretic peptide shown as geometric mean (95% CI); eGFR = estimated glomerular filtration rate based on serum creatinine and cystatin C levels [28]. CKD defined as eGFR < 60 ml/min/1.73m^2^.

**Table 2.** Risk factor characteristics by Chronic Kidney Disease (CKD) status and by heart failure status in the Multi-Ethnic Study of Atherosclerosis

Variables	CKD Group (n=325)			Non-CKD Group (n=5,179)		
	HF (n=39)	No HF (n=286)	P value	HF (n=180)	No HF (n=4,999)	P value
Age (years)	73.3±7.0	72.9±8.1	0.8	67.9±8.7	61.4±10.0	<0.001
Sex (Male)	64.1%	40.2%	0.005	60.0%	48.5%	0.002
BMI (kg/m^2^)	29.6±5.6	28.4±5.4	0.2	29.5±5.6	28.2±5.4	<0.001
Smoking	5.1%	8.7%	0.6	13.9%	12.8%	0.7
SBP (mmHg)	144±26.8	139±24.7	0.16	137±21.9	125±20.8	<0.001
Heart rate (bpm)	62.7±10.7	63.8±11.3	0.6	65.9±10.5	63.0±9.5	<0.001
Diabetes	35.9%	19.6%	0.02	31.7%	12%	<0.001
+NT-proBNP (pg/mL)	331 (215, 510)	143 (127, 161)	<0.001	126 (106, 149)	45.6 (44.2, 47.0)	<0.001
eGFR (mL/min/1.73m^2^)	46.6±12.2	49.2±10.8	0.2	86.2±15.0	92.6±15.5	<0.001

Data presented as mean ± SD or frequency (%) unless otherwise noted. Abbreviations: CKD = chronic kidney disease; HF = heart failure; BMI = body mass index; SBP = systolic blood pressure; eGFR = estimated glomerular filtration rate based on serum creatinine and cystatin C levels [28]; NT-proBNP = N-terminal pro-brain natriuretic peptide; CKD defined as eGFR < 60 ml/min/1.73m^2^;

+ P values calculated by comparing mean values of log transformed NT-proBNP levels and values shown as geometric means (95% CI).

**Table 3.** Cox regression risk analysis for prediction of heart failure by Chronic Kidney Disease (CKD) status in the Multi-Ethnic Study of Atherosclerosis (n=5504).

	Univariate		Multivariable	
	HR (95% CI)	P Value	HR (95% CI)	P Value
Variable	Chronic Kidney Disease (eGFR < 60 ml/min/1.73 m^2^) (n=325)			
Age (years)	1.01 (0.97, 1.06)	0.5	1.01 (0.96, 1.06)	0.8
Sex (Male)	2.67 (1.39, 5.14)	<0.001	3.14 (1.58, 6.25)	0.001
BMI (kg/m^2^)	1.04 (0.98, 1.10)	0.2	1.05 (0.99, 1.12)	0.1
Smoking status	0.65 (0.16, 2.71)	0.6	0.58 (0.13, 2.66)	0.5
SBP (mmHg)	1.01 (0.997, 1.02)	0.1	1.01 (0.99, 1.02)	0.4
Heart rate (bpm)	0.99 (0.97, 1.02)	0.7	1.01 (0.98, 1.04)	0.6
Diabetes	2.41 (1.25, 4.64)	0.01	1.56 (0.72, 3.35)	0.3
logNT-proBNP (pg/mL)	2.12 (1.63, 2.74)	<0.001	1.90 (1.47, 2.46)	<0.001
*eGFR (mL/min/1.73m^2^)	0.97 (0.95, 0.99)	0.04	0.99 (0.97, 1.02)	0.5
Variable	No Chronic Kidney Disease (eGFR ^≥^ 60 ml/min/1.73 m^2^) (n=5,179)			
Age (years)	1.08 (1.06, 1.09)	<0.001	1.04 (1.02, 1.06)	<0.001
Sex (Male)	1.62 (1.20, 2.18)	0.002	2.68 (1.97, 3.66)	<0.001
BMI (kg/m^2^)	1.04 (1.02, 1.07)	0.001	1.05 (1.02, 1.08)	<0.001
Smoking status	1.15 (0.75, 1.75)	0.5	1.59 (1.03, 2.48)	0.04
SBP (mmHg)	1.02 (1.02, 1.03)	<0.001	1.01 (0.999, 1.01)	0.1
Heart rate (bpm)	1.03 (1.02, 1.05)	<0.001	1.04 (1.02, 1.05)	<0.001
Diabetes	3.53 (2.58, 4.83)	<0.001	2.42 (1.74, 3.37)	<0.001
logNT-proBNP (pg/mL)	2.42 (2.11, 2.78)	<0.001	2.36 (2.04, 2.73)	<0.001
*eGFR (mL/min/1.73m^2^)	0.97 (0.96, 0.98)	<0.001	0.99 (0.98, 1.004)	0.2

Data presented as Hazard Ratios (95% CI). Abbreviations: CKD = chronic kidney disease; eGFR= estimated glomerular filtration rate calculated with serum creatinine and cystatin C; BMI = body mass index; SBP = systolic blood pressure; logNT-proBNP = log transformed N-terminal pro-brain natriuretic peptide.

* eGFR and log transformed NT-proBNP were tested separately, and multivariable HRs and p values for other variables are from a model which included log transformed NT-proBNP and not eGFR

**Table 4.** Measures of model fit for prediction of heart failure with heart failure risk variables (HFRV) only or combined with estimated glomerular filtration rate (eGFR), N-terminal pro-brain natriuretic peptide (NT-proBNP) or both among adults with and without Chronic Kidney Disease in the Multi-Ethnic Study of Atherosclerosis (n=5504).

	HFRV	HFRV + eGFR	HFRV + NT-proBNP	HFRV + eGFR + NT-proBNP
Chronic Kidney Disease (eGFR < 60 ml/min/1.73 m^2^) (n=325)				
C-statistic (95% CI)	0.71 (0.64, 0.78)	0.73 (0.64, 0.78)	0.78 (0.71, 0.85)*	0.79 (0.71, 0.86)*
Log likelihood	−204.2	−204.0	−193.4	−192.1
Akaike Information Criterion	422.4	424.0	402.8	402.3
Bayes Information Criterion	448.8	454.2	433.0	436.3
Net Reclassification Index	Referent	6.53%	23.1%	28.8%*
Risk Stratification Capacity	64.3%	63.1%	77.8%	77.2%
No Chronic Kidney Disease (eGFR ≥ 60 ml/min/1.73 m^2^)(n=5,179)				
C-statistic (95% CI)	0.77 (0.74, 0.80)	0.77 (0.74, 0.80)	0.83 (0.80, 0.85)*	0.83 (0.80, 0.85)*
Log likelihood	−1420.4	−1419.6	−1357.6	−1357.5
Akaike Information Criterion	2854.9	2855.2	2731.2	2733.1
Bayes Information Criterion	2900.7	2907.6	2783.6	2792.0
Net Reclassification Index	Referent	−0.46%	10.2%*	15.9%*
Risk Stratification Capacity	96.1%	96.2%	97.7%	96.8%

Abbreviations: eGFR= estimated glomerular filtration rate calculated with serum creatinine and cystatin C. [28] Heart failure risk variables (HFRV) = age, sex, body mass index, smoking, systolic blood pressure, heart rate, and diabetes.

* P < 0.05 compared with model with heart failure risk variables (HFRV) only

**Table 5.** Reclassification table for events and nonevents comparing heart failure risk variable (HFRV) model with model adjusting for HFRV, log N-terminal pro-brain natriuretic peptide (logNTproBNP), and estimated glomerular filtration rate (eGFR) among adults with CKD

		HFRV + logNTproBNP + eGFR model			
**HFRV model**	Events (n=39)	<5%	5–10%	>10%	Row Total
	<5%	2	2	0	4
	5–10%	8	8	11	27
	>10%	1	0	7	8
	Column Total	11	10	18	39
		HFRV + logNTproBNP + eGFR model			
**HFRV model**	Non-Events (n=286)	<5%	5–10%	>10%	Row Total
	<5%	135	16	2	153
	5–10%	52	30	7	89
	>10%	8	18	18	44
	Column Total	195	64	27	286

Abbreviations: HFRV = heart failure risk variables (age, sex, body mass index, smoking, systolic blood pressure, heart rate, and diabetes); logNTproBNP = log transformed N-terminal brain natriuretic peptide; eGFR = estimated glomerular filtration rate; CKD = chronic kidney disease. Each interior cell contains the number of persons in the corresponding risk categories under the old and new risk models. Corresponding NRI = 28.8% (p=0.02; p_events_=0.40; p_non-events_<0.001) in CKD group.

**Table 6.** Reclassification table for events and nonevents comparing heart failure risk variable (HFRV) model with model adjusting for HFRV, log N-terminal pro-brain natriuretic peptide (logNTproBNP), and estimated glomerular filtration rate (eGFR) among adults without CKD

		HFRV + logNTproBNP + eGFR model			
**HFRV model**	Events (n=180)	<5%	5–10%	>10%	Row Total
	<5%	122	20	7	149
	5–10%	5	6	12	23
	>10%	3	2	3	8
	Column Total	130	28	22	180
		HFRV + logNTproBNP + eGFR model			
**HFRV model**	Non-Events (n=4,999)	<5%	5–10%	>10%	Row Total
	<5%	4,682	92	15	4,789
	5–10%	109	39	30	178
	>10%	9	9	14	32
	Column Total	4,800	140	59	4,999

Abbreviations: HFRV = heart failure risk variables (age, sex, body mass index, smoking, systolic blood pressure, heart rate, and diabetes); logNTproBNP = log transformed N-terminal brain natriuretic peptide; eGFR = estimated glomerular filtration rate; CKD = chronic kidney disease. Each interior cell contains the number of persons in the corresponding risk categories under the old and new risk models. Corresponding NRI=15.9% (p<0.001; p_events_<0.001; p_non-events_=0.54) in non-CKD group.
